# Visualization of the field of knowledge in sexual violence: a scientometric analysis based on citespace

**DOI:** 10.5249/jivr.v16i1.1862

**Published:** 2024-01

**Authors:** Ali Hamidi, Abdolrasool Khosravi, Roghayeh Hejazi, Fatemeh Torabi, Allahkaram Akhlaghi

**Affiliations:** ^*a*^ Department of Medical Library and Information Sciences, Bushehr University of Medical Sciences, Bushehr, Iran.; ^*b*^ Department of Knowledge and Information Science, Faculty of Educational Sciences and Psychology, Shahid Chamran University, Ahvaz, Iran.; ^*c*^ Faculty of Management and Information, Iran University of Medical Sciences, Tehran, Iran.

**Keywords:** Sex Offenses, Research/trends, Scientometric, Biblioshiny, Citespace

## Abstract

**Background:**

Sexual violence (SV) is a serious public health problem affecting millions of people each year. The main aim of this article is to provide a large-scale snapshot of the field of knowledge in SV research using a scientometric approach.

**Methods:**

Documents were retrieved from the Web of Science database. Then, a scientometric study was carried out on a sample of 65,610 documents. Co-citation and co-occurrence measures have been calculated and related networks have been drawn using Citespace and Biblioshiny software.

**Results:**

The main findings indicate that research in SV has increased significantly in recent years. On the other hand, the publication of about one-third of these documents by a single author is due to the special nature of this topic and its taboo in many societies. In addition, a large number of multimedia documents demonstrate the role and importance of multimedia resources in SV studies. Despite the attention to SV research by poor or developing countries to research in the field of SV, 95% of the documents have been published by 20 developed countries. Additionally, the general research approach has changed from criminology to psychology.

**Conclusions:**

Therefore, it seems that the discussion of psychological disorders in the occurrence of sexual violence reveals a new approach to SV. The concepts related to SV have been linked to broader areas than in the past. This, along with emphasizing prevention topics in the long term, will in-crease awareness of SV and reduce the possibility of abuse of vulnerable people.

## Introduction

A Sexual violence (SV) is a serious public health problem affecting millions of people each year.^1^ The economic burden of SV is high in both high and low-income countries. The direct costs of medical care, law enforcement, and lost productivity are substantial.^2,3^ There are also indirect costs associated with the long-term physical and mental health consequences of sexual violence.^4,5,6,7^ In addition, the COVID-19 pandemic has intensified the risk of increasing aspects of sexual violence in various societies^8,9^ and led researchers to pay more attention to this issue.^10^

SV refers to the coercion of sexual activity without consent,^11^ such as sexual assault, rape, and sexual abuse. In SV, the victim is unable to consent (e.g., because of age or illness) or to refuse (e.g., because of physical violence or threats).^12^

Anyone can experience SV, including children, ado-lescents, adults, and the elderly.^11^ According to the WHO,^13^ one-quarter of adults report having been physically abused as a child, one in five women report having been sexually abused as a child, and one in three women have been the victim of physical SV or SV by a sexual partner at some point in their lives. SV can take the form of rape or sexual assault, child sexual assault and incest, intimate partner sexual assault, unwanted sexual contact/touching, sexual harassment, sexual ex-ploitation, exposing one’s genitals or a naked body to others (s) without consent, masturbating in public, and watching someone in a private act without their knowledge or permission.^11^

A review of the literature on SV showed that research in this area has increased in recent years.^14,15,16^ However, the studies that have been conducted to determine the overall status of SV research have usually focused on one or more aspects of SV or have been limited to a specific geographic location.^13,14,17,18,15,16,19^ Therefore, due to the large volume of literature in this area, a detailed study of the overall state of research on SV is needed. The study of scientific articles as a research output is very relevant as an object of analysis because it reflects lines, trends, and research potentials in research communities.^20^ Scientometrics is one of the most appropriate methods to know the general situation of a subject area. Using scientometric techniques, we can discover the relationship between institutional characteristics at the level of research groups and developments at the level of disciplines and scientific specialties.^21^ The results of this research help researchers in the field to know the subject areas of the articles, the level of cooperation, people, and institutions in the field, which can be useful in choosing research topics and potential collaborators. Therefore, the main goal of this article is to provide a large snapshot of SV research using the scientometric approach, including co-occurrence and cocitation networks.

## Methods

Scientometric analysis refers to the use of quantitative methods to examine the literature of a field.^21^ In this approach, statistical tools are used to map developments and evaluate the published works of a subject area. Content analysis field, in addition to scientometric analysis, content analysis is used to examine the literature of a subject area and future horizons by examining the co-occurrence of words in the target texts of interest.^22^ Therefore, the scientometric approach was used in this research. In this method, citation and communication networks are drawn by using different indicators such as co-occurrence, cocitation, and coauthorship.

**Data collection**

The first step in scientific research is data collection. Since citation data provides links between different documents, it is necessary to collect data from citation databases. The Web of Science database is one of the most reliable databases providing such data. Therefore, this database was selected as the data collection source. Considering the different aspects of SV studies, all the keywords related to SV were identified by studying the related texts and using MeSH.

In the initial search, we understood that the keyword rape, which is one of the most important keywords in sexual violence, is also considered an oil seed. Therefore, we tried to remove irrelevant documents by limiting the search by removing the subject categories of agriculture and those related to oil seeds. In this way, documents that used the keyword rape in nonsexual concepts were not included in the retrieved collection. Finally, the following search string was performed on February 22, 2023, and 65,610 documents were retrieved. Then, in the Export section, the full record and cited references were saved in the format of.txt files for further analysis.

(TS= (Sex* NEAR/3 Offens*) OR TS=(Sex* NEAR/3 Assault*) OR TS=(Sex* NEAR/3 Violence*) OR TS=(Sex* NEAR/3 Abuse*) OR TS=(rape) OR TS=("Gender*Based violence*") NOT (TS=(rapeseed OR “Brassica napus” OR napus* OR cabbage* OR Brasicaceae OR canola* OR oil* OR wheat* OR "sugar beet*" OR leave* OR herbicid* OR polymorph* OR farm* OR arable* OR corn* OR grow* OR pollen* OR pollinat* OR seed* OR pesticide* OR plant* OR soil* OR agriculture* OR agronom* OR horticultur* OR entomolog*)

**Analysis**

In this study,scientometric were examined at the level of authors, journals, articles, organizations, and countries. Descriptive information was mainly extracted from the Biblioshiny software (Aria & Cuccurullo, 2017), which was developed in the R language. Because it is based on the R programming language, Biblioshiny is highly flexible and easy to use. Citespace software (Chen, 2006; Chen et al., 2012) was used to draw co-occurrence and collaboration networks. In addition, the capabilities of this software allow drawing co-authorship, co-occurrence, and cocitation networksin addition, this software, was used to calculate indicators such as node centrality, network density, modularity, and cluster silhouette of clusters in the network.

## Results

**Descriptive information**

Table 1 shows the descriptive information of the retrieved data. Due to the time limitations of the database that covered documents from 1945, the documents retrieved between 1945 and 2023 were published in 8688 sources by 107539 authors with an annual growth rate of 7.63%. The noteworthy point in this table is the average age of the documents, which is reported as 12.8 year, indicating the dominance of new documents over older ones. A total of 20,421 single-authored documents have been published, which includes approximately 31% of the documents. This shows the specificity of the investigated area considering the smaller share of single-authored documents in other field (citations). In the document type section, the variety of documents, especially those related to multimedia sources, shows the role and importance of multimedia sources in SV studies.

**Table 1:** Main descriptive information of retrieved data.

Description	Results	DOCUMENT TYPES	Results
MAIN INFORMATION ABOUT THE DATA		Article	48755
Timespan	1945:2023	article; book chapter	1967
Sources (Journals, Books, etc.)	8688	article; data paper	6
Documents	65610	article; proceedings paper	1233
Annual Growth Rate %	7.63	article; retracted publication	2
Document Average Age	12.8	book	252
Average citations per doc	23.08	book review	3206
References	1258470	correction	154
DOCUMENT CONTENTS		including reviews(including dance perfor-mance, film, music performance, record, software, theater, tv, radio, art exhibit and video)	3609
Keywords Plus	24623	discussion	17
Author's Keywords	49952	editorial material	1936
AUTHORS		fiction, creative prose	12
Authors	107539	letter	1008
Authors of single-authored docs	15032	meeting abstract	1984
AUTHORS COLLABORATION		news item	157
Single-authored documents	20421	note	355
Co-Authors per document	3.16	poetry	32
International coauthorships %	12.4	proceedings paper	805
		review	3562

In the following, coauthorship networks, cooperation of countries, and cooperation of organizations have been analyzed.

**Coauthorship networks**

**Figure 1:**
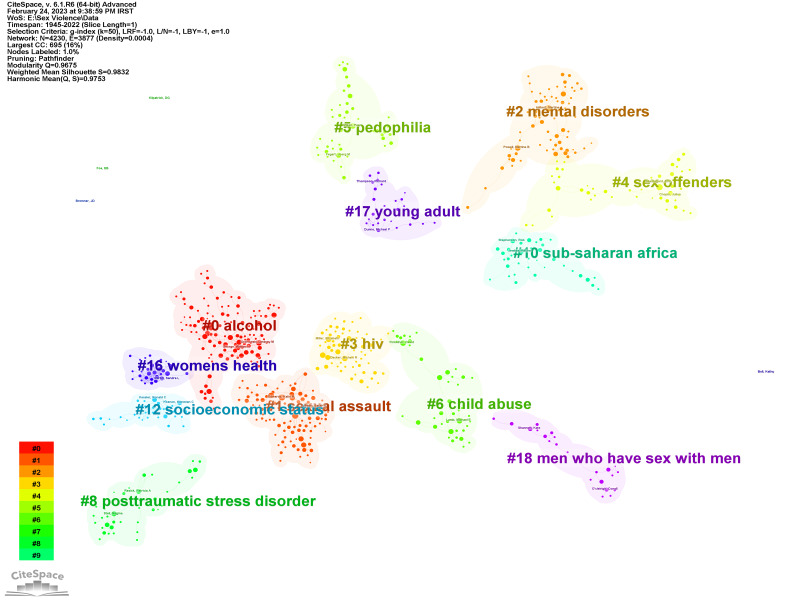
Coauthorship network.

The first analysis includes the co -authorship network (Figure 1). This network with a density of 0.0004 is a loose network. in addition, the harmonic Mean (Q, S) = 0.9735 shows that the cooperation between authors in this field was low. The main clusters in this network include alcohol, sexual assault, mental disorders, HIV, sex offenders, and pedophilia. These clusters indicate the important topics for collaboration between authors.

**Figure 2:**
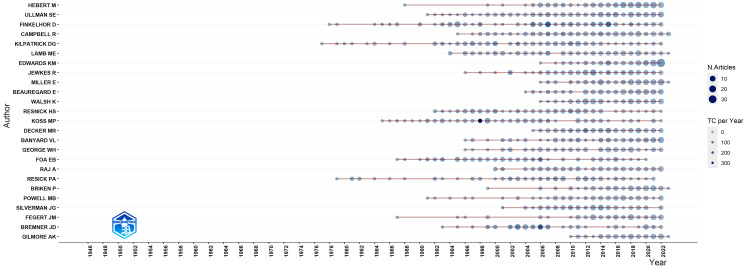
Most prolific authors.

Figure 2 shows the most prolific authors in SV. The activity of the oldest and newest of these authors started in 1978 and 2006, respectively. However, almost all of these authors are still active and continue their research.

**Organizations’ collaboration network**

**Figure 3:**
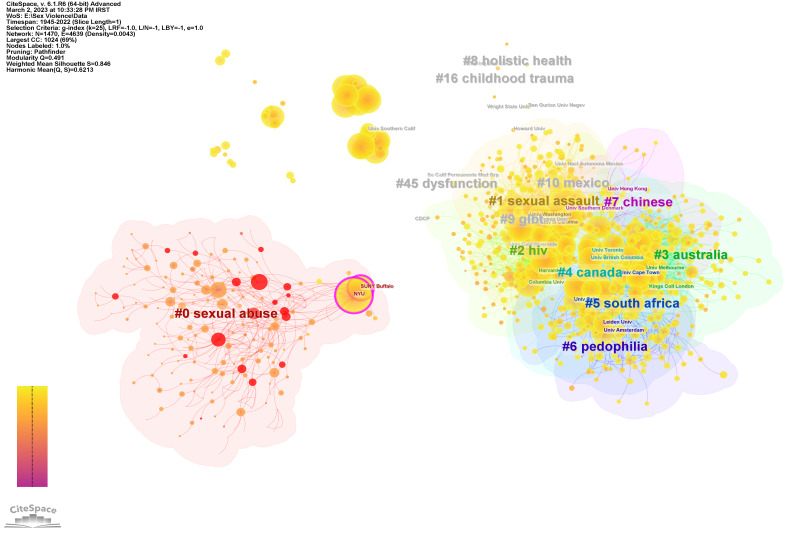
Organizations' collaboration network.

Figure 3 shows the network of collaboration between organizations. In this network, based on the number of citations, the most productive organizations are the University of Washington, Harvard University, University of Toronto, University of North Carolina, and Columbia University, with 739, 702, 693, 987, and 674 documents, respectively. Since the nodes with centrality above 0.1 are considered nodes that shape or reshape the network structure, the most effective organizations in terms of shaping the network structure are New York University, Suny Buffalo, and the University of South California (Table 2 ). The noteworthy point is that all three universities are placed in cluster #0, and the average year of its members is 1985. Therefore, it can be said that this relatively old cluster play a crucial role in shaping research and collaboration between organizations. Topics discussed in this cluster include sexual abuse, chronic pain, childhood physical abuse, prepubertal girls, vaginal washing, child abuse, substance abuse, sexual assault, and women's health.

**Table 2:** The most influential organizations based on centrality measures.

Centrality	Organization	Cluster-ID
0.16	New York University	0
0.12	University at Buffalo	0
0.07	University of Southern California	0
0.04	National Institute of Mental Health	2
0.04	University of Michigan	0
0.04	Yale University	0
0.04	McLean Hospital	0
0.03	Harvard University	2
0.03	Emory University	1
0.03	University of Oxford	3

**Country collaboration network**

**Figure 4:**
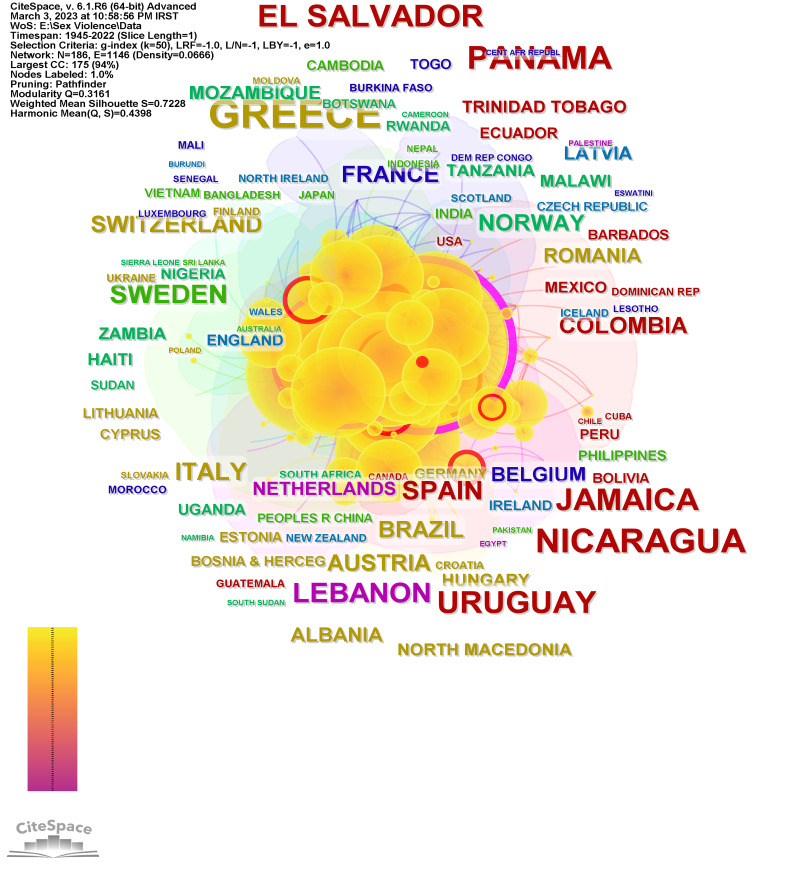
Country collaboration network.

Figure 4 shows the collaboration network of cooperation between countries. This network with modularity Q=0.3161 is a network with considerable overlap. The remarkable aspect of this figure is the presence of countries that are classified as poor countries in international rankings and that have many problems in the area of SV. However, the most influential countries in this network are the United States, the United Kingdom, and France, with centralities of 0.53, 0.18, and 0.11, respectively.

**Keyword Co-occurrence Network**

**Figure 5:**
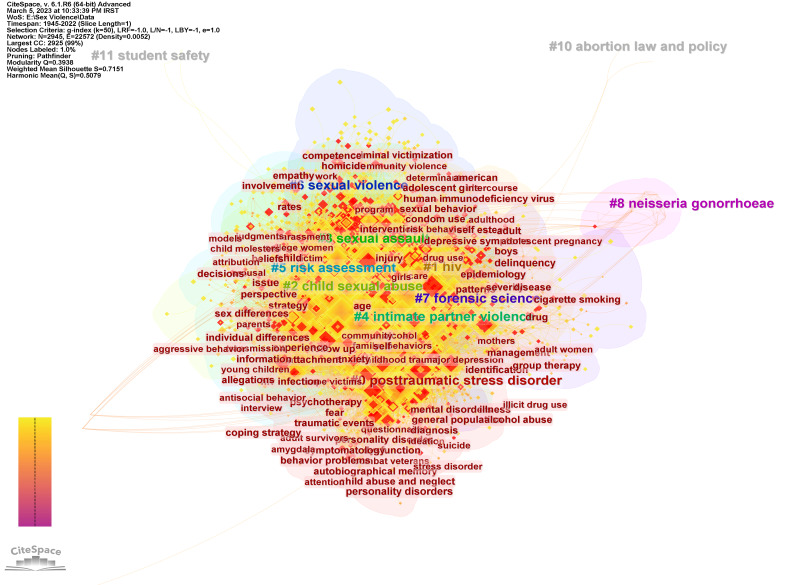
Keywords' co-occurrence network.

By analyzing the co-occurrence of keywords, it is possible to identify topics of interest to researchers are identified in different time periods. According to the data in Table 3 , except for cluster 8 with 12 members and average year of activity in 1990, the average year of activity of other clusters is referred to after 2003. Thus, the topics discussed in cluster 8 include sexual assault, specific antigens, enzyme immunoassay, p-30 glycoprotein, seminal acid phosphatase chlamydia trachomatis, sexuality transmitted diseases, sexual abuse, and Neisseria gonorrheae. On the other hand, the topics discussed in cluster 6 with an average activity year of 2014 include sexual violence, night economy, emotional abuse, nursing students, gender-neutral toilets, gender-based violence, scoping review, East Africa, and service delivery. It should be noted that the newest cluster is Cluster 11, with an average year of activity of 2019, but due to the small size of this cluster (3 members), its data are not reliable (Figure 5).

**Table 3:** Keywords' co-occurrence clusters information.

Cluster	Size	Silhouette	Mean Year			
0	685	0.667	2003	...	posttraumatic stress disorder (1538.97, 1.0E-4); ptsd (885.99, 1.0E-4); trauma (694.83, 1.0E-4); childhood trauma (620.57, 1.0E-4); sexual violence (557, 1.0E-4)	gonozyme(r) (1.58); gono-chek ii (1.58); schools (1.58); antigen detection test (1.58); role of media (1.58)
1	430	0.695	2005	sexual abuse; posttraumatic stress disor-der; birth weight; perinatal outcomes; perinatal self-care | intimate partner violence; female sex workers; viral load; availability; medical cost	hiv (1022.46, 1.0E-4); sexually transmit-ted infections (416.35, 1.0E-4); infection (372.93, 1.0E-4); condom use (338.16, 1.0E-4); sex work (317.3, 1.0E-4)	gonozyme(r) (0.97); gono-chek ii (0.97); schools (0.97); antigen detection test (0.97); role of media (0.97)
2	390	0.691	2003	sexual abuse; forensic interviews; victims; investigative interviews; suggestibility | child abuse; drug therapy; behavioral therapy; emotional abuse; nuisance crimes	child sexual abuse (1677.75, 1.0E-4); suggestibility (471.55, 1.0E-4); memory (460.92, 1.0E-4); testimony (312.71, 1.0E-4); forensic interviews (308.3, 1.0E-4)	gonozyme(r) (0.94); gono-chek ii (0.94); schools (0.94); antigen detection test (0.94); role of media (0.94)
3	379	0.715	2005	sexual abuse; forensic interviews; victims; investigative interviews; suggestibility | child abuse; drug therapy; behavioral therapy; emotional abuse; nuisance crimes	sexual assault (1815.99, 1.0E-4); rape (1060.41, 1.0E-4); sexual aggression (614.13, 1.0E-4); aggression (554.12, 1.0E-4); college students (545.36, 1.0E-4)	gonozyme(r) (1.79); gono-chek ii (1.79); schools (1.79); antigen detection test (1.79); role of media (1.79)
4	369	0.748	2003	sexual abuse; forensic interviews; victims; investigative interviews; suggestibility | child abuse; drug therapy; behavioral therapy; emotional abuse; nuisance crimes	intimate partner violence (567.2, 1.0E-4); child maltreatment (543.52, 1.0E-4); sex-ual abuse (527.43, 1.0E-4); rape (473.68, 1.0E-4); physical abuse (468.98, 1.0E-4)	gonozyme(r) (2.64); gono-chek ii (2.64); schools (2.64); antigen detection test (2.64); role of media (2.64)
5	321	0.713	2009	sexual abuse; risk assessment; sexual offenders; sexual recidivism; serial sex | sex offenders; juvenile sex offenders; sexual deviance; executive functioning; relapse	risk assessment (1018.6, 1.0E-4); recidi-vism (999.08, 1.0E-4); sex offenders (621.75, 1.0E-4); sex offender (535.33, 1.0E-4); sexual offending (517.89, 1.0E-4)	gonozyme(r) (0.38); gono-chek ii (0.38); schools (0.38); antigen detection test (0.38); role of media (0.38)
6	203	0.772	2014	sexual violence; night-time economy; emotional abuse; students nursing; gen-der-neutral toilets | gender-based vio-lence; scoping review; east africa; ser-vice provision; night-time economy	sexual violence (1883.62, 1.0E-4); gen-der-based violence (635.09, 1.0E-4); war (634.78, 1.0E-4); human rights (574.02, 1.0E-4); violence against women (526.27, 1.0E-4)	gonozyme(r) (0.45); gono-chek ii (0.45); schools (0.45); antigen detection test (0.45); role of media (0.45)
7	129	0.89	2008	forensic science; sexual assault evidence; forensic biology; clinical forensic medicine; rape victim examination | sexual assault; sperm hy-liter; sperm detection; fluores-cence staining; paternal identification	forensic science (716, 1.0E-4); drug-facilitated sexual assault (318.55, 1.0E-4); ghb (258.14, 1.0E-4); dna (206.42, 1.0E-4); benzodiazepines (197.81, 1.0E-4)	gonozyme(r) (0.09); gono-chek ii (0.09); schools (0.09); antigen detection test (0.09); role of media (0.09)
8	12	0.997	1990	sexual assault; specific antigen; enzyme immu-noassay; p-30 glycoprotein; seminal acid phosphatase | chlamydia trachomatis; trans-mitted diseases; sexual abuse; neisseria gon-orrhoeae; remote areas	neisseria gonorrhoeae (33.36, 1.0E-4); en-zyme immunoassay (33.36, 1.0E-4); gonozyme(r) (16.64, 1.0E-4); gonochek ii (16.64, 1.0E-4); antigen detection test (16.64, 1.0E-4)	sexual abuse (0.03); sexual assault (0.03); sexual vio-lence (0.02); rape (0.02); intimate partner violence (0.02)
9	4	1	2001	abortion law; unsafe abortion; reproductive health services; service providers; sexual violence | sexual violence; political process; unsafe abortion; service providers; abor-tion law	abortion law and policy (54.45, 1.0E-4); sexual vio-lence and abuse (36.14, 1.0E-4); mexico (19.7, 1.0E-4); role of media (17.99, 1.0E-4); clandestine and unsafe abortion (17.99, 1.0E-4)	sexual abuse (0.03); sexual as-sault (0.03); sexual violence (0.02); rape (0.02); intimate part-ner violence (0.02)
10	3	1	2019	school personnel; student safety; sexual abuse; sexual misconduct; school employee	student safety (35.31, 1.0E-4); school employee sexual misconduct (35.31, 1.0E-4); school personnel (35.31, 1.0E-4); schools (17.59, 1.0E-4); standard of care (17.59, 1.0E-4)	sexual abuse (0.03); sexual assault (0.03); sexual violence (0.02); rape (0.02); intimate partner violence (0.02)

**Main Clusters**

**Figure 6:**
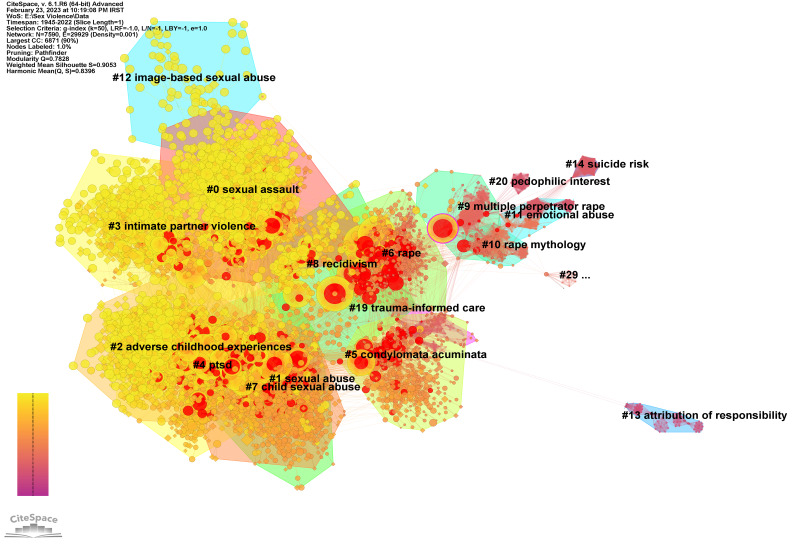
Documents' cocitation network.

To identify the main clusters, the cocitation network of documents was drawn by selecting documents as nodes (Figure 6). This network has distinct clusters with modularity Q=0.7828 and weighted average silhouette S=0.9053, showing that the nodes in each cluster are homogeneous. The density of 0.001 also indicates the formation of relatively good connections between the nodes studied. Table 4 shows that cluster #9 is the oldest cluster formed with an average age of 1937. The main topics discussed in this cluster include crime scene analysis, behavioral theme, smallest space analysis, offender profiling, male victims, and risk assessment, which shows the emphasis of old articles in the field of SV on criminological topics.

**Table 4:** Documents' cocitation main titles.

Cluster	Size	Silhouette	Mean Year	LSI	LLR	MI
0	1097	0.902	2007	sexual assault; adult victims; sexual dysfunction; sex thera-py; crime reporting | sexual violence; sexual minority; sub-stance use; women's health; night-time economy	sexual assault (1158.59, 1.0E-4); rape (492.29, 1.0E-4); sexual violence (368.57, 1.0E-4); rape myths (280.77, 1.0E-4); by-stander intervention (202.63, 1.0E-4)	perpetrator status (1.74); genital hpv infections (1.74); consciousness-raising (1.74); behavioral theme (1.74); children sports (1.74)
1	1020	0.876	1994	sexual abuse; eating disorders; young women; sexual abuse characteristics; dissociative ex-periences | child abuse; cluster analysis; family background; learning theory; young women	sexual abuse (358.96, 1.0E-4); sexual violence (147.25, 1.0E-4); sexual assault (138.6, 1.0E-4); childhood sexual abuse (133.93, 1.0E-4); intimate partner vio-lence (105.65, 1.0E-4)	perpetrator status (0.67); genital hpv infections (0.67); consciousness-raising (0.67); behavioral theme (0.67); children sports (0.67)
2	988	0.875	2008	sexual abuse; primary preven-tion; sibling aggression; emo-tional abuse; disordered eating attitudes | child abuse; social support; sibling aggression; emotional abuse; disordered eating attitudes	sexual assault (440.02, 1.0E-4); adverse childhood experiences (439.68, 1.0E-4); child maltreat-ment (328.77, 1.0E-4); childhood maltreatment (311.75, 1.0E-4); childhood trauma (265.86, 1.0E-4)	perpetrator status (1.88); genital hpv infections (1.88); consciousness-raising (1.88); behavioral theme (1.88); children sports (1.88)
3	730	0.94	2008	intimate partner violence; sub-saharan africa; interparental violence; interpersonal violence; lgbq college students | domestic violence; sexual assault; child abuse; cultural contexts; inter-personal violence	intimate partner violence (1297.82, 1.0E-4); domestic violence (644.08, 1.0E-4); child sexual abuse (233.31, 1.0E-4); dating violence (208.01, 1.0E-4); physical violence (185.83, 1.0E-4)	perpetrator status (1.41); genital hpv infections (1.41); consciousness-raising (1.41); behavioral theme (1.41); children sports (1.41)
4	720	0.895	2002	posttraumatic stress disorder; sexual abuse survivors; affect-management group therapy; general practitioner; animal models | sexual abuse; trau-matic events; violent behavior; posturography; immobility	PTSD (827.21, 1.0E-4); post-traumatic stress disorder (644.26, 1.0E-4); trauma (379.71, 1.0E-4); post-traumatic stress disorder (207.29, 1.0E-4); hippocampus (183, 1.0E-4)	perpetrator status (1.13); genital hpv infections (1.13); consciousness-raising (1.13); behavioral theme (1.13); children sports (1.13)
5	608	0.9	1977	sexual abuse; child perpetrator; abuse-specific effects; preven-tion education; family systems | child abuse; forensic medicine; abuse reporting; family sys-tems; interview processes	sexual abuse (82.33, 1.0E-4); sexual assault (30.54, 1.0E-4); condylomata acuminate (27.94, 1.0E-4); sexually transmitted diseases (27.94, 1.0E-4); ana-tomical dolls (25, 1.0E-4)	perpetrator status (0.05); genital hpv infections (0.05); sexuality development (0.05); teacher education (0.05); behavioral (0.05)
6	555	0.88	1978	sexual assault; rape culture; focal concerns; victim reporting; cluster | sexual violence; online abuse; sex-shaming; revenge porn; online victim	rape (98.41, 1.0E-4); rape myths (49.42, 1.0E-4); child abuse (24.17, 1.0E-4); sexual abuse (23.86, 1.0E-4); rape myth acceptance (20.24, 1.0E-4)	domestic violence offenders (0.09); criminal offender specialization (0.09); mindful space (0.09); evidence standardization (0.09); internet pornography (0.09)
7	307	0.945	2001	sexual abuse; repressed memory; betrayal trauma theo-ry; traumatic amnesia; sex ex-change | child abuse; sex ex-change; emotional abuse; social perceptions; criminal trials	child sexual abuse (320.2, 1.0E-4); disclosure (300.78, 1.0E-4); forensic interviews (122.51, 1.0E-4); forensic interviewing (105.64, 1.0E-4); investigative interviews (104.13, 1.0E-4)	perpetrator status (0.29); genital hpv infections (0.29); consciousness-raising (0.29); behavioral theme (0.29); children sports (0.29)
8	244	0.956	2000	sexual abuse; catholic church; post-traumatic stress disorder treatment; cyproterone ace-tate; demographic characteris-tics | risk assessment; post-traumatic stress disorder treat-ment; cyproterone acetate; demographic characteristics; father-daughter sex abuse	recidivism (386.35, 1.0E-4); risk assessment (363.52, 1.0E-4); pedophilia (306.26, 1.0E-4); sex offender (256.99, 1.0E-4); sex offenders (222.87, 1.0E-4)	perpetrator status (0.29); genital hpv infections (0.29); consciousness-raising (0.29); behavioral theme (0.29); children sports (0.29)
9	155	0.977	1937	crime scene analysis; behavioral theme; smallest space analysis; offender profiling; on-male sexual assault | stranger rape; offender penetration; interper-sonal interaction; male victims; risk assessment	multiple perpetrator rape (28.66, 1.0E-4); behavioral theme (14.29, 0.001); dyads (14.29, 0.001); crime scene analysis (14.29, 0.001); offend-er profiling (14.29, 0.001)	sexual assault (0.04); sexual abuse (0.03); child sexual abuse (0.02); intimate part-ner violence (0.02); sexual violence (0.02)

On the other hand, clusters #0, #2, #3, #4, #7, and #8, whose average year of activity is after 2000, deal with psychological concepts such as sexual dysfunction, sibling aggression, emotional abuse, intimate partner violence, posttraumatic stress disorder (PTSD), violent behavior, and emotional abuse. Therefore, it seems that in these clusters, the discussion of psychological disorders in the occurrence of sexual violence reveals a new approach to SV. The interesting point among these clusters is cluster #6, which has an average of 1978 and includes topics such as online abuse and online victims.

## Discussion

Sexual violence is a pervasive problem that affects individuals and communities around the world. Defined as any behavior of a sexual nature that causes the victim to feel discomfort, fear, or worry, sexual violence can take many forms, including rape, molestation, harassment, and assault. One of the main concerns is that SV is a serious problem that can have long-term consequences for both the victim and the perpetrator. It refers to the behavior of a carnal or emotional nature that causes the victim discomfort, fear, or anxiety. It is an unfortunate and widespread phenomenon that has been observed across cultures, age groups, and genders.^12^ Given the impact that SV can have on the lives and futures of victims and even perpetrators, many studies have been conducted in this area. In the search conducted through the Web of Science database, 65,610 related documents were retrieved between 1945 and 2023. The growth rate of 7.63% and the average lifespan of 12.8 years indicate the increasing importance and serious attention of the scientific community to this issue, especially in recent years. On the other hand, the fact that one-third of these documents are published as single-authored documents is due to the special nature of this topic and its taboo in many societies. However, the importance and pervasiveness of this issue and the higher probability of victimization of the weaker section of society, including women and children, has made the use of multimedia resources to publish research results more significant than other issues.

Our research showed that 107,539 authors are active in this field, the most prolific of which started in 1978. The important point about prolific authors is that these authors are still active and continue to research regardless of the year they started their activity. This shows the commitment and interest of active researchers in the field.

Findings related to influential organizations show that most of these organizations operate in the United States. The issue of interest to these organizations focuses on women's health and issues related to children. According to active organizations, women's health and child abuse are the most important issues in the area of SV.

The network of collaborating countries shows that despite the influence of the United States, England, and France, poor countries play an important role in conducting research in this field. This can be justified considering the high prevalence of SV in poor and less developed countries. In other words, it seems that research on SV in poor countries is considered necessary. However, 95% of documents have been published by 20 countries that are considered developed countries. Therefore, despite the activity and influence of poor countries in this area, the volume of this activity does not seem sufficient, and the policymakers of these countries as well as international organizations need to have appropriate plans in this regard.

The keyword co-occurrence network shows that, except for one cluster (#8), the average activity year of cluster activity is after 2003. Topics discussed in the oldest cluster include sexual assault-specific antigen, enzyme immunoassay, p-30 glycoprotein, seminal acid phosphatase|chlamydia trachomatis, transmitted diseases, sexual abuse, and Neisseria gonorrhea. Since these topics are related to the diagnosis and consequences of SV and especially rape, the emphasis of old research is criminology. Examining the main clusters of the cocitation network also confirms this finding. In other words, the main topics in the oldest cluster of this network are crime scene analysis, behavioral theme, smallest space analysis, offender profiling, male victims, and risk assessment, which emphasize criminology. On the other hand, other clusters that contain newer topics include issues such as PTSD, intimate partner violence, female sex workers, emotional abuse, nuisance crimes, child abuse, abortion, and student safety. In this case, the concepts raised in the cocitation network of documents are consistent with the co-occurrence network of keywords. The main concepts in the document cocitation network include sexual dysfunction, sibling aggression, emotional abuse, intimate partner violence, posttraumatic stress disorder, violent behavior, and emotional abuse.

Based on the findings, it is clear that the volume of research on SV has increased significantly in recent years in terms of volume and will probably increase significantly in the coming years. In addition, the general research approach has changed from criminology to behavior modification, prevention of SV, and new areas of SV, such as emotional abuse and online abuse. In this way, it is clear that the concepts related to SV have been linked to broader areas than in the past. This, together with the emphasis on prevention issues, will in the long term, increase awareness of SV and will reduce the possibility of abuse of weaker people.

**Limitations**

This study used the Web of Science database was used to collect data. The emphasis of this database on English-language studies and the limited scope of its indexing are considered limitations of this research. In addition, the possibility of not retrieving part of the related data can be considered as another limitation of this research.
